# Validation of the Colombian–Spanish Suicidality Scale for Screening Suicide Risk in Clinical and Community Settings

**DOI:** 10.3390/jcm13247782

**Published:** 2024-12-20

**Authors:** Ana María Arenas Dávila, Katherine Pastrana Arias, Óscar Mauricio Castaño Ramírez, Pamela Van den Enden, Juan Carlos Castro Navarro, Santiago González Giraldo, Doris Mileck Vera Higuera, Keith M. Harris

**Affiliations:** 1 Department of Mental Health, Universidad de Caldas, Manizales 170004, Colombia; k-tpa@hotmail.com (K.P.A.); oscar.castano@ucaldas.edu.co (Ó.M.C.R.); pamelavandenenden@gmail.com (P.V.d.E.); jcnmdpsi@gmail.com (J.C.C.N.); santiago.gonzalez@ucaldas.edu.co (S.G.G.); doris.vera@ucaldas.edu.co (D.M.V.H.); 2 Independent Researcher, Brisbane 4073, Australia; esensei@yahoo.com

**Keywords:** validation studies, suicide prevention, suicide, clinical decision making, Colombia, translations

## Abstract

**Background/Objective**: This study aimed to validate the eight-item Suicidality Scale (SS) in Spanish in a Colombian sample to aid in suicide risk assessment, given the pressing need for accurate, accessible tools in resource-strained settings. **Methods**: A sample of 313 participants, drawn from both clinical and community settings, was used to evaluate the psychometric properties of the SS through tests of internal consistency, item response theory (IRT), and comparisons with clinical risk evaluations. **Results**: The SS demonstrated strong psychometric properties, with high internal consistency (ω = 0.96) and a significant correlation with clinical risk assessments (r = 0.84). Model fit indices confirmed a unidimensional eight-item structure with low error rates, while item response analysis revealed strong item discrimination. No differential item functioning was observed by gender or psychiatric diagnosis, supporting its consistency across demographics. Items on past suicide attempts and desire to live were excluded as they did not improve scale performance. Variability within risk levels suggests that individual differences may require clinical judgment. **Conclusions**: The findings validate the Colombian–Spanish SS as a valuable tool for suicide risk assessment, usable in both self-report and clinician-administered formats. Its brief, culturally adapted structure supports its utility in resource-limited environments, providing an accessible option for rapid screening. While the SS effectively categorizes general risk, further longitudinal studies are recommended to enhance its applicability in guiding clinical decisions and long-term risk management.

## 1. Introduction

Suicide stands as one of the most critical global public health challenges, demanding urgent efforts to develop reliable tools for early detection and timely intervention [1]. Each year, suicide accounts for 1.5% of all deaths worldwide [2], disproportionately affecting vulnerable populations and developing regions where mental health resources are often limited [3].

In Colombia, where suicide rates are 7.5 per 100,000 for women and 13.7 per 100,000 for men [4,5], prevention efforts are significantly hindered by limited access to mental healthcare, particularly in rural areas [6]. Additionally, the pervasive influence of socioeconomic stressors on mental health, including intramarital conflicts and financial difficulties, is exacerbated by the country’s ongoing issues with violence and economic instability [7,8,9,10]. Addressing these challenges requires culturally adapted, accessible, and psychometrically sound tools that align with the UN Sustainable Development Goals, advancing equitable healthcare and reducing global mental health inequalities [11].

Several instruments are used in Colombia to assess suicide risk, including the Scale for Suicide Ideation (SSI) [12], the Self-Rated Scale for Suicide Ideation (Sr-SSI) [13], the Modified Scale for Suicide Ideation (MSSI) [14], the SAD PERSONS Scale [15], and the Plutchik Suicide Risk Scale [16]. Among these, only the Plutchik Suicide Risk Scale has been validated within the Colombian context [17]. However, existing tools often present challenges, including excessive length, the need for specialized training, or potential inaccuracies in risk estimation, making them impractical in clinical settings.

Accurately assessing suicidal intent is crucial for evaluating both immediate and long-term risk of suicide. To date, the accuracy of suicide prediction models is inadequate [7]. To improve this situation, it is helpful to be guided by well-validated psychological theories, such as attitude formation. The tripartite model of attitudes (e.g., toward suicide or death) comprises three correlated but distinct components: affect, behavior, and cognition [18,19]. Evidence has shown that these are useful factors to measure the risk of current and future suicidal behaviors. The affective component encompasses hopelessness the wish to live (WTL) and the wish to die (WTD), which are considered part of a “motivational dimension” in suicidality [20,21,22,23]. The relationship between suicidality and affect is supported by mixed evidence. Cognitive aspects, such as death-related ideation and suicidal thoughts, are also predictors of suicide risk [23,24,25,26,27,28].

Behavioral components, such as past suicide attempts, are considered predictors of suicidality, though there is mixed evidence regarding their predictive strength. While some studies present past attempts as very strong predictors, others do not find an association that is as robust. Additionally, non-suicidal self-harm (NSSH) has not been shown to provide additional predictive value when included in models that already account for suicidal cognition and behaviors [27,29,30,31,32,33,34,35,36,37,38]. Suicidality, viewed as a latent trait, reflects the interplay of these dimensions.

The Suicidal Affect–Behaviors–Cognition Scale (SABCS) is grounded in the tripartite model. Developed using item response theory (IRT), the SABCS assesses suicidality as a unidimensional construct encompassing affective, behavioral, and cognitive dimensions. It has demonstrated high internal consistency and strong construct validity, and has been validated across diverse populations [39].

Based on its strong psychometrics, we aimed to validate the SABCS to assess suicide risk in Hispanic populations. However, the six-item SABCS was recently part of a large psychometric project, which resulted in the development of the eight-item Suicidality Scale (SS) [39], including items on past suicide attempts and WTL. The SS studies showed that the behavioral items did not function well across broad and diverse samples. Only a behavioral intention item—on the possibility of future suicide attempts—demonstrated strong validity for inclusion in a clinically useful risk assessment. The final eight-item SS demonstrated strong psychometric properties across ages 13–80, genders, and other demographic variables, with no evidence of differential item functioning (DIF).

The present study aimed to test the validity of the SS in Hispanic communities and psychiatric samples in Colombia. After a critical evaluation of the ensemble of items and their relevance for the local population, the decision was made to develop and validate a culturally and Spanish language-adapted suicide risk assessment (SRA) based on the SS. As a distinct construct, the resulting eight-item Spanish Suicidality Scale (S-SS) struck a balance between being a brief, easy-to-administer tool and being sufficiently precise to capture key elements of suicide risk without overwhelming the evaluator with additional questions. The S-SS will be made freely available through Creative Commons BY (free culture) licensing.

## 2. Materials and Methods

### 2.1. Ethics and Open Science

This study complied with the ethical standards outlined by national and institutional human experimentation committees and the Helsinki Declaration of 1975, revised in 2008. Ethical approval was granted by the Ethics Committee of Universidad de Caldas (ACTA No 003 de 2021, consecutive number CBCS-007). The research further adhered to Colombian guidelines for human research [40] and the World Medical Association’s Declaration of Helsinki [41].

Aligned with open science principles and the United Nations Sustainable Development Goals [11], this study aims to promote equitable access to scientific findings. The data and analysis code are openly accessible at https://osf.io/fze6j/ (accessed on 22 July 2023).

Funding was provided by the Territorial de Salud de Caldas, which did not influence the study’s design, implementation, or reporting, thereby ensuring no conflicts of interest.

### 2.2. Recruitment and Administration

Participants were recruited in person between March and June 2021. After providing informed consent, they completed the SS-S and demographic questions. The self-report questionnaire required less than five minutes, and no incentives were provided for participation.

All participants underwent semi-structured psychiatric evaluations conducted by licensed psychiatrists or psychiatric residents. They were classified as “diagnosed” if they met the criteria for at least one mental health disorder according to the DSM-5 criteria, while those who did not were categorized as “non-diagnosed.” If, during the assessment, any participant was identified as being at risk of suicide, regardless of their psychiatric diagnosis, they were referred for further evaluation by the team to determine the necessary interventions.

Privacy was protected through the assignment of unique identifiers.

### 2.3. Design and Procedure

**Figure 1:**
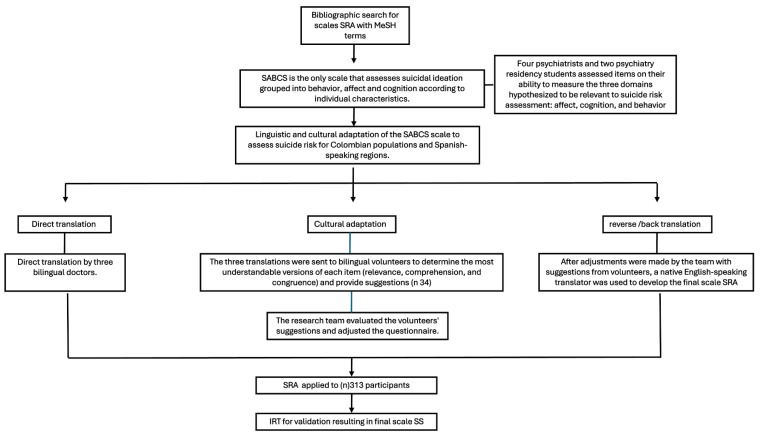
Study design and procedure flowchart. The methodological framework for validating the SS (Suicidality Scale) [39].

This study used a cross-sectional descriptive design to validate the SRA tailored to the Colombian population. As outlined in Figure 1, the methodological framework encompassed scale selection, translation, cultural adaptation, and validation through community and clinical testing. Data collection included the administration of the SRA and a comprehensive evaluation of psychometric properties.

### 2.4. Translation and Adaptation

The SRA included the eight-item SS and two supplementary items. It was translated and culturally adapted to ensure relevance for Spanish-speaking communities.

A committee of six mental health experts (four psychiatrists and two psychiatry residents) evaluated the SRA, composed of the eight-item SS and two supplementary items (history of suicide attempts and wish to live), for their ability to measure affect, cognition, and behavior.

Direct translations were performed by three bilingual clinicians and refined based on feedback from 34 volunteers, who rated item clarity and validity. Suggestions were incorporated, and the final questionnaire underwent back-translation by a native-English-speaking translator. We similarly verified the linguistic equivalence to Colombian–Spanish. A sample of over 300 participants was selected for initial validation, following [42].

Development of the SS found that suicidal behaviors were not of sufficient value for assessing current risk at the population level. Nevertheless, the authors recommended including behavior items for supplementary information. The SS demonstrated strong psychometric properties and validity across individuals with English as a first or second language, gender, and those ages 13–80. The SS can be applied by clinicians, people without clinical training, or can be self-administered and includes descriptions of suicidality levels based on IRT analyses. For this study, we included the eight cognitive and affective SS items, plus two supplementary items: past suicide attempts (Attempt), ranging from none to “at least once really wanted to die”; and wish to live (WTL), a reverse-scored polar opposite to the wish to die (WTD) item included in the SS. We aimed to critically examine the validity of all items, including supplementary items, for inclusion in a final Spanish SS. See Appendix A for total items and responses.

### 2.5. Clinical Ratings

Evaluations for clinical participants were conducted by licensed psychiatrists and psychiatry residents who classified participants into predefined suicide risk categories based on gatekeeper training protocols and professional expertise. Among the 313 participants, 1.6% were categorized as ultra-high risk, 14.1% as high risk, 13.1% as moderate risk, 19.5% as low risk, and 55.3% as having no identifiable risk.

### 2.6. Analyses

We used the R psych package to conduct a multimodel approach to evaluate the SRA items and scale critically. Analyses included minimum residual factor analysis (FA, direct oblimin rotation), which produces an unweighted least squares solution that is robust for skewed data [43]. This included common variance, item communalities (h2), factor loadings, and model fit (Tucker–Lewis Index, TLI; root mean square error of approximation, RMSEA). Hierarchical cluster analysis and bifactor analysis also included model fit, error, and item loadings. We calculated McDonald’s omega (ω), bootstrapped 1000 iterations, as a robust estimate of internal consistency for congeneric scales [44]. For IRT analyses, we used the graded response model [45,46,47]. Analyses were conducted with the open-source R statistical environment, v.4.2.0 [48] including the following packages: ltm [49], lordif [50], and coefficient alpha [51].

We also tested item monotonicity and linearity through rest-score plots [52]. Graded response model (GRM) analyses also led to creating individual person scores derived from empirical Bayesian estimates [49]. These IRT-derived theta values are based on unique item characteristics and response patterns. In contrast, traditional sum scores assume all items are equally weighted and all item steps are equivalent. We examined invariance by demographics (e.g., age, sex, residence) and clinical factors (psychiatric diagnosis) through differential item functioning (DIF). DIF is recommended over confirmatory factor analysis (CFA) and other invariance tests [53]. The lordif package uses GRM modeling to detect evidence of DIF through R^2^ change (≥0.02).

## 3. Results

The SRA, including the SS along with the clinical evaluation, was administered to all 313 participants. Demographic characteristics of the participants included an age range of 18–65 years (M = 35.29), with 42% female and 58% male. The sample was composed of single people, followed by smaller proportions who were married, cohabitating, separated, divorced, or widowed. The majority of participants identified as Mestizo (nearly 95%), with smaller groups identifying as White, Mulatto, Indigenous, or Afro-Colombian. About four out of five participants lived in urban areas, while the remainder resided in rural settings. Socioeconomic status was predominantly concentrated in the middle–lower range, with nearly three-quarters falling within social strata 2 and 3 on Colombia’s six-tier classification system.

### 3.1. Psychometric Properties and Scale Reliability

The SS included eight core items assessing suicidality and two supplementary items—Attempt and wish to live. Table 1 shows that core items demonstrated consistent variability and near-normal distributions, supporting their reliability. In contrast, WTL and Attempt showed greater variability and deviation from normality, with WTL displaying moderate skewness and kurtosis and Attempt contributing less to scale reliability. SS person scores demonstrated lower skewness and kurtosis. Individual standard residuals ranged from −0.87 to 2.87 and can be found in the data file with the other data at https://osf.io/fze6j/ (accessed on day 22 July 2023).

We examined the psychometric properties of all ten SRA items. Table 2 contains the final SS item diagnostics of our four-step analyses. First, we tested the general unidimensionality and suitability of the items through these four different psychometric models, with a critical view of each item’s specific properties. The analyses can be considered to be in order of sophistication, with cluster analyses providing general item and cluster properties and GRM providing the most advanced information. However, each test contributes unique and meaningful results that are useful for testing and confirming scale validity. Hierarchical cluster analysis showed high loadings across the eight final items and provided support for a unidimensional item set. Next, factor analysis (minimum residual) confirmed strong common factor loadings, a unidimensional item set, and strong commonalities, with one exception. RFD showed a somewhat low communality (<0.60) but was still moderately strong. Bifactor analyses further supported a unidimensional model, with high loadings and communalities and no evidence of meaningful subgroup factors.

**Figure 2:**
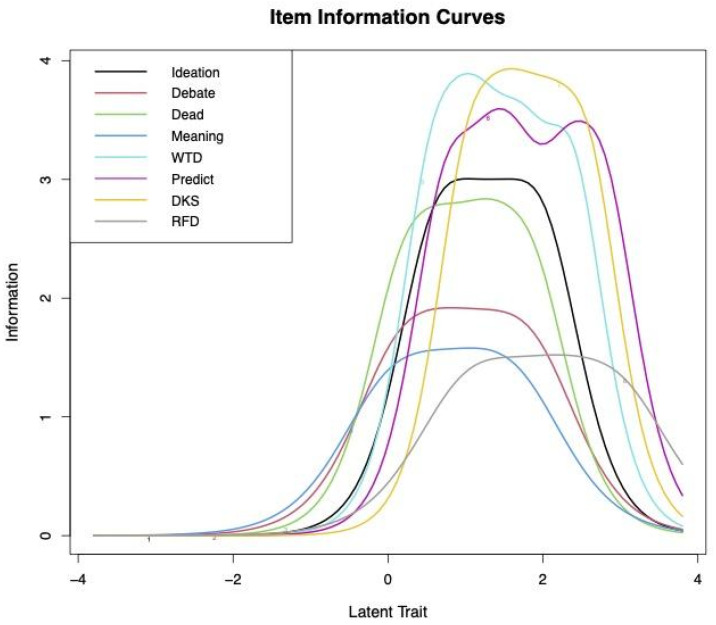
Suicidality Scale item characteristic curves.

GRM results showed that all items had high discrimination, other than Attempt. For WTL, discrimination was moderately high but below 2.0. These results indicate that the Attempt item does not make a sufficient contribution to measuring suicidality and that WTL is somewhat weaker than other items. We next conducted rest-score plots and found a lack of linearity between WTLr and other items, demonstrating that this item is related to the latent trait but inconsistent with others. Figure 2 shows the GRM item characteristic curves, illustrating the b values, with the area under each line representing the item’s information function (the amount of information on the latent trait that that item provides).

### 3.2. Construct Validity

Exploratory factor analysis confirmed the unidimensionality of the SS. A single-factor solution accounted for 68.5% of the variance in suicidality, with most items achieving acceptable factor loadings (≥0.71). The Attempt item fell below acceptable thresholds, supporting its limited utility. High communalities further supported the scale’s ability to encapsulate suicidality as a unified latent construct.

We further tested for group invariance to determine whether the SS exhibited any bias across groups. The results showed no differential item functioning (DIF) for gender or psychiatric diagnosis (ΔR^2^ < 0.02). Due to sample size and distribution limitations, DIF analyses for other variables were not conducted. Instead, we examined the SS across the full sample and by age groups. As shown in Table 3, diagnostics were nearly identical; all fit indices were very high, and error was reasonably low. All conditions similarly exhibited strong internal consistency.

The SS performed well for younger age groups but showed lower factorability and higher error for participants over 50 years. This is likely due to the small subsample size but warrants further review.

### 3.3. Clinical and Test Evaluations

**Figure 3:**
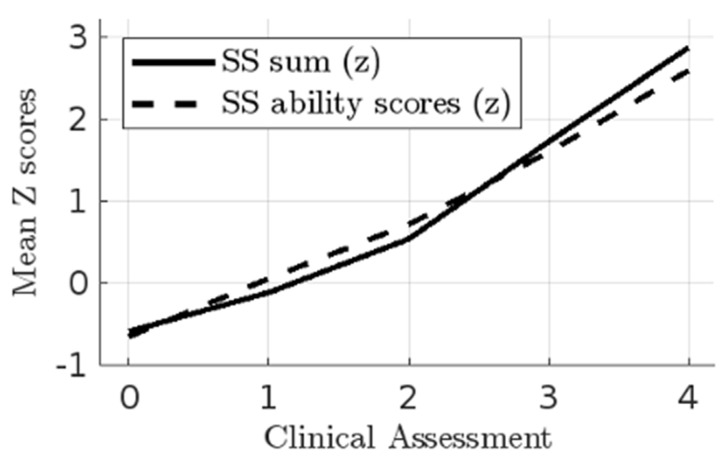
Correlations between clinical and Suicidality Scale assessments.

We then compared SS person scores and sum scores with clinical decisions for these participants. First, we tested for demographic confounds, finding small but statistically significant associations between SS scores with sex and age, with males and younger participants reporting higher suicidality (*ps* < 0.05). Bootstrapped partial correlations (controlling for sex and age) showed strong correlations between clinical assessments, made on a five-point ordered categorical scale, with SS sum scores with r = 0.82, 95% CI [0.78, 0.87] and SS person scores with r = 0.84, 95% CI [0.79, 0.88]. However, Figure 3 shows some important variance; clinical ratings matched sum scores and person scores at the non-risk and highest risk levels. However, clinical ratings appear to better match the person scores than the sum scores at the low to moderate risk levels.

## 4. Discussion

Following the adaptation and field testing of Colombian–Spanish suicide risk assessment questions, we developed a concise, evidence-based Spanish Suicidality Scale (S-SS) designed for use in Hispanic communities. The SS has been validated across diverse populations, demonstrating strong psychometric properties in both clinical and community settings, as well as across different demographic groups and psychiatric diagnoses. Clinical evaluations showed strong correlations with S-SS scores while also identifying some important distinctions. These results highlight the S-SS’s potential to guide clinical decision making by focusing on the most prevalent symptoms of suicidality, supported by this and additional evidence.

The S-SS offers several benefits. It can be used as a self-report and it does not require prior specialized training for application and interpretation, allowing it to be used in a variety of settings. However, an improved understanding of interpreting latent trait measures would be helpful across the mental health field. The S-SS is briefer, at eight items, compared to other tests. For example, the Self-Rated Scale For Suicide Ideation—SRSSI—includes 19 items [13]; the Modified Scale For Suicide Ideation—MSSI—has 18 items [14]; and the Plutchik scale, which is the only SRA previously validated in Colombia, has 26 items. This parsimony makes it more favorable for assessing risk in an emergency or primary care service where there is little time per consultation [17]. The SS and S-SS present as a unique construct, evaluating three dimensions of suicide: affect (e.g., Wish to die), behavioral intentions (Predict), and cognition (e.g., Debate). In addition, other scales (e.g., SADPERSONS) apparently overestimate suicide risk, leading to the hospitalization of patients who do not require it [15,54].

To make the best use of our data, we utilized IRT and bifactor analysis, and conducted DIF. These analytical approaches provide deeper insights into the latent trait under investigation [55]. IRT-derived person (ability) scores enable precise identification of an individual’s position on the underlying suicidality continuum. Additionally, these methods help determine which questions yield the most informative responses, allowing for greater emphasis on those items when assessing suicide risk.

We tested ten items, but after reviewing multimodel analyses, we found that items related to previous suicide attempts and WTL provided less information on the latent trait of suicide. These items were not invalid, but they did not perform well across a broad sample. There are likely two different issues here. First, suicidal behaviors are rare and, therefore, most people respond “no” to such items, while those who respond “yes” may have attempted or planned suicide years ago but are now mentally healthy and not suicidal. That reduces the benefit of such an item other than for important biographical information. Secondly, reverse-scored items sometimes fail to function well. The WTL item showed weakness across our psychometric analyses, similar to that of the larger SS study [39]. It could be that WTL is capturing information on a trait that is related but distinct to suicidality.

However, the suicide attempts item, in particular and along with a suicide plans item, can provide valuable additional biographic data. While that data can aid the understanding and treatment of patients, those items did not demonstrate sufficient validity for inclusion in scale scores. Once removed, the fit indices for the 8-item version were higher than for the 10-item version, showing that their removal contributes to a better fit of the model data, consistent with the English and Chinese studies [39].

It was notable that the clinician ratings corresponded better to the IRT-derived person scores than to sum scores. There was strong agreement at the lowest and highest risk levels, but it seems clinicians are picking up on additional information to make their decisions at low to moderate risk levels. That appears to offer some validation for the accuracy of person scores over the simple summing of all items.

### Limitations

There are important limitations to this study, such as sample size and the need for testing mid- and long-term suicidal outcomes through longitudinal study. However, the greatest limitation may be in deciding how patients should be placed into outcome categories, ranging from immediate discharge to emergency care. SS cutoff scores (e.g., low risk, moderate risk, or high risk) are very appealing, and many scales include them. However, this study and all known studies on suicide measurement have demonstrated that such cutoff values are built on false assumptions, as scale items demonstrate varying weights, discrimination levels, etc. There are, fortunately, highly useful means for making the most of these instruments.

Scores should serve as a guide rather than dictate clinical decisions. The S-SS is most effective in a comprehensive evaluation, with scores interpreted along a spectrum. Low scores generally suggest low or no suicidality, high scores indicate severe risk necessitating immediate intervention, and intermediate scores call for careful clinical judgment. This approach avoids rigid reliance on arbitrary cutoffs while leveraging the scale’s strengths. Furthermore, the self-report S-SS is designed to be accessible, eliminating the need for literacy or specialized training in interviewer-administered protocols, making it suitable for use in developing regions. Importantly, the clinician-administered SS has proven effective, with no reported issues.

## 5. Conclusions

The S-SS showed strong psychometrics and represents a step forward in accurate risk assessment for adults and diverse populations. Importantly, the S-SS was developed through community involvement as a localized instrument. It is not enough to create a high-quality tool; local knowledge and skills are required for sustainable use and future development. The S-SS is free culture licensed, allowing for free use and modification. This can aid communities and individuals who require high-quality risk assessments but who have limited resources.

The S-SS is a valuable tool for monitoring therapeutic progress. Administering the scale at multiple intervals allows clinicians to track changes in risk levels following psychotherapeutic or pharmacological interventions. This functionality supports tailored adjustments to care and provides insights into treatment effectiveness. While more longitudinal studies are needed to assess the scale’s utility in shaping treatment directions, its dual role in initial risk assessment and ongoing evaluation can enhance clinical outcomes and promote sustained mental health recovery.

Our study suggests that the SS-S should be evaluated in additional demographic subgroups and compared with other suicide instruments validated in Central and South America. It is also important to study suicide risk assessment in younger age groups. Despite the fact that suicide risk is a very difficult health factor to assess, with the validation of this scale, we come a little closer to understanding this mental health problem.

## Figures and Tables

**Table 1:** Suicidality Scale and item characteristics (N = 313).

Item/Scale	Range	*M*	*SD*	Skew	Kurtosis
DKS	1–6	1.59	1.25	2.16	6.63
WTD	1–6	1.89	1.57	1.58	4.09
Dead	1–6	2.16	1.76	1.26	3.03
Debate	1–6	2.72	1.79	1.11	2.73
Ideation	1–6	1.95	1.61	1.55	4.05
Predict	1–6	1.63	1.26	2.14	6.69
Meaning	1–6	2.33	1.84	1.06	2.57
RFD	1–6	1.59	1.23	2.19	6.89
SS sum	8–48	15.41	10.91	1.49	1.08
SS person	−0.80–3.08	0.10	1.04	0.94	−0.17
WTLr	1–6	1.88	1.55	1.57	4.06
Attempt	1–4	1.75	1.19	1.17	2.58

Note: SE skew (SES) = 0.14; SE kurtosis = 0.28. DKS = desire to kill self; WTD = wish to die; RFD = reason for dying; sum = sum score; person = graded response model-derived person score; SS = Suicidality Scale; WTLr = wish to live, reverse-scored.

**Table 2:** Colombian–Spanish Suicidality Scale item characteristics.

Item	Clus	FA		BA		GRM		
		L	*h* ^2^	g	*h* ^2^	LL	UL	a
Dead	0.91	0.91	0.83	0.87	0.90	0.29	1.69	3.68
Ideation	0.85	0.86	0.73	0.87	0.92	0.52	1.83	3.49
Debate	0.86	0.86	0.73	0.83	0.77	0.17	1.64	3.22
Predict	0.88	0.87	0.75	0.84	0.90	0.77	2.47	2.48
DKS	0.88	0.87	0.76	0.83	0.85	0.93	2.49	3.55
Meaning	0.85	0.85	0.72	0.81	0.81	0.07	1.47	3.13
WTD	0.91	0.92	0.84	0.87	0.83	0.60	2.05	3.57
RFD	0.77	0.77	0.59	0.72	0.63	1.00	2.91	2.80

Note: Clus = hierarchical cluster analysis; FA = minimum residual factor analysis; BA = bifactor analysis (Schmid–Leiman); L = common factor loading; g = general factor; *h*^2^ = communalities; DKS = desire to kill self; WTD = wish to die; RFD = reason for dying.

**Table 3:** Colombian–Spanish Suicidality Scale diagnosis across ages.

Scale	Cluster		FA			BA			ω	95% CI
	Fit	RMSR	V	TLI	RMSEA	ω^h^	ECV	RMSEA		
SS	0.98	0.05	0.74	0.88	0.19	0.89	0.84	0.08	0.96	[0.95, 0.96]
Age1	0.98	0.05	0.76	0.85	0.22	0.92	0.84	0.20	0.96	[0.95, 0.97]
Age2	0.98	0.06	0.75	0.83	0.23	0.87	0.80	0.03	0.96	[0.95, 0.96]
Age3	0.96	0.09	0.67	0.62	0.32	0.76	0.65	0.26	0.94	[0.56, 0.98]

Note: FA = minimum residual factor analysis; BA = bifactor analysis; RMSR = root mean square of residuals; TLI = Tucker–Lewis Index of factoring reliability; RMSEA = root mean square error of approximation; V = variance; **ω^h^** = general factor variance; ECV = explained common variance; ω = internal consistency, bootstrapped 1000 iterations. SS = Suicidality Scale; Age1 = 18–25 (n = 93); Age2 = 26–49 (n = 158); Age3 = 50–84 (n = 64).

## Data Availability

The original contributions presented in the study are included in the article, further inquiries can be directed to the corresponding author/s.

## Appendix A

ESCALA COLOMBIANA DE RIESGO DE SUICIDIO

Nos gustaría hacerte algunas preguntas personales relacionadas con el suicidio. Por favor respóndelas de la manera más precisa posible.

PREGUNTA 1: ¿Con qué frecuencia has pensado en suicidarte durante el último año?

- Nunca (1) (2) (3) (4) (5) (6) Muy frecuentemente.

PREGUNTA 2: Recientemente, ¿has tenido una discusión interna (en tu cabeza) acerca de vivir o morir?

- Nunca (1) (2) (3) (4) (5) (6) Muy Frecuentemente.

PREGUNTA 3: Últimamente, ¿has tenido pensamientos acerca de que estarías mejor muerto(a)?

- Nunca. (1) (2) (3) (4) (5) (6) Muy Frecuentemente.

PREGUNTA 4: ¿Recientemente, has tenido el sentimiento de que tu vida no tiene sentido?

- Nunca (1) (2) (3) (4) (5) (6) Muy Frecuentemente.

PREGUNTA 5: Recientemente, ¿qué tanto has deseado morir?

- No deseo para nada morir (1) (2) (3) (4) (5) (6) Mucho.

PREGUNTA 6: ¿Qué tan probable es que en el futuro cercano intentes suicidarte?

- No hay ninguna probabilidad (1) (2) (3) (4) (5) (6) Es muy probable.

PREGUNTA 7: En una escala del 1 al 6 siendo 1 la ausencia de deseos de suicidarte, y 6 un deseo intenso de hacerlo ¿dónde te ubicas?

- No tengo deseos de suicidarme (1) (2) (3) (4) (5) (6) Tengo un deseo intenso de suicidarme.

PREGUNTA 8: En una escala del 1 al 6 evalúa, ¿qué tantas razones tienes para VIVIR o MORIR?

- Tengo más razones para VIVIR que para morir (1) (2) (3) (4) (5) (6) Tengo más para MORIR que para vivir.
